# A Cubesat Payload for Exoplanet Detection

**DOI:** 10.3390/s17030493

**Published:** 2017-03-02

**Authors:** Marcella Iuzzolino, Domenico Accardo, Giancarlo Rufino, Ernesto Oliva, Andrea Tozzi, Pietro Schipani

**Affiliations:** 1 Department of Industrial Engineering—Aerospace Division, University of Naples Federico II, 80125 Naples, Italy; domenico.accardo@unina.it (D.A.); giancarlo.rufino@unina.it (G.R.); 2 INAF—Astrophysical Observatory of Arcetri, Largo E. Fermi 5, 50125 Florence, Italy; oliva@arcetri.astro.it (E.O.), atozzi@arcetri.astro.it (A.T.); 3 INAF—Astrophysical Observatory of Capodimonte, Salita Moiariello, 16, 80131 Naples, Italy; pietro.schipani@oacn.inaf.it

**Keywords:** exoplanets, cubesat, photometric transit, photometry, pyramid, false positive

## Abstract

The search for undiscovered planets outside the solar system is a scientific topic that is rapidly spreading into the astrophysical and engineering communities. In this framework, the design of an innovative payload to detect exoplanets from a nano-sized space platform, like a 3U cubesat, is presented. The selected detection method is photometric transit, and the payload aims to detect flux decrements down to ~0.01% with a precision of 12 ppm. The payload design is also aimed at false positive recognition. The solution consists of a four-facets pyramid on the top of the payload, to allow for measurement redundancy and low-resolution spectral dispersion of the star images. The innovative concept is the use of a small and cheap platform for a relevant astronomical mission. The faintest observable target star has V-magnitude equal to 3.38. Despite missions aimed at ultra-precise photometry from microsatellites (e.g., MOST, BRITE), the transit of exoplanets orbiting very bright stars has not yet been surveyed photometrically from space, since any observation from a small/medium sized (30 cm optical aperture) telescope would saturate the detector. This cubesat mission can provide these missing measurements. This work is set up as a demonstrative project to verify the feasibility of the payload concept.

## 1. Introduction

An exoplanet is a planet that is orbiting around a star other than the Sun. An Earth-like planet is a habitable planet of approximately 1 Earth mass and 1 Earth radius, and in an Earth-like orbit around a Sun-like star at a distance of roughly 1 AU [1].

By now (February 2016) the number of total confirmed exoplanets (From http://exoplanetarchive.ipac.caltech.edu/. The criteria to list an exoplanet as confirmed are as follows: the mass is equal or less than 30 Jupiter masses; the planet is associated with a host star (not free floating); sufficient follow-up observations and validation have been undertaken to deem that the object being a false positive is unlikely; the above information along with further orbital and/or physical properties are available in peer-reviewed publications) is 3449. Among these, the number of planets discovered by the photometric transit method is 2718 (From http://exoplanetarchive.ipac.caltech.edu/), showing that this method is very effective. Of those 2718 planets, the number of terrestrial planets, with planet mass between 0.6 Earth mass and 10 Earth masses, is 80 (From http://exoplanetarchive.ipac.caltech.edu/).

The photometric transit technique consists of measuring the reduction of the star photometric flux when the planet passes in front of the host star as seen from the Earth. In order to realistically observe the exoplanet transit, the exoplanet orbit around the host star must be aligned with the observer. The measurement of the photometric star flux as a function of time is depicted in the so-called light curve of the target star.

The objective of detecting exoplanet transit is becoming a priority among the current astronomical space missions. The following is a brief list of the more relevant past/present/future missions regarding exoplanet transit detection.

CoRoT is the first mission ever designed to detect exoplanet transit from space, led by ESA and by CNES (Centre national d’études spatiales) from 2006 to 2013 [2]. The 27 cm diameter telescope observed in the band from 370 nm to 950 nm, and a one facet prism was installed before the CCDs used for transit detection [3]. Kepler is the NASA’s first mission (from 2009 to 2013) able to find Earth-sized planets around Sun-like stars. The 0.95 m diameter telescope has a FOV (field of view) of 105 deg^2^, an array of 42 CCDs observing in the wavelength band from 400 nm to 850 nm, with instrument precision of 10^−5^. It continuously points at a single star field in the Cygnus-Lyra region, with a large number of stars but off the galactic plane to reduce field confusion [4]. TESS is the first spaceborne all-sky exoplanet transit survey from NASA-MIT (Massachusetts Institute of Technology), scheduled in 2017, observing in the band from 600 nm to 1000 nm [5]. CHEOPS is the first small class mission from ESA (European Space Agency), scheduled in 2017, to detect exoplanet transit on stars with V-magnitude between six and twelve anywhere in the sky, observing in the band from 0.4 to 1.1 µm [6]. PLATO is a medium class exoplanet transit detection mission from ESA (scheduled in 2024) in orbit around L2, looking at stars with a V-magnitude between 4 and 16 [7].

There are also cubesat projects that aim at astronomical missions regarding exoplanet detection; a brief project list is provided in the following.

The most relevant cubesat project is the pioneer Exoplanetsat, a 3U cubesat from MIT and DraperLab. As a pathfinder mission, the mission objective is to detect exoplanet transit around bright stars with V-magnitude less than four. The key aspect of this project is a fine target pointing achieved by a closed control loop on the detector position to compensate for the satellite jitter [8]. The project evolved in ASTERIA, a 6U cubesat with improved photometric capabilities. MDOT is a 6U cubesat project from Stanford University in a HEO (High Earth Orbit) orbit. The project aims at taking a direct image of exo-zodiacal dust and transiting exoplanets using an occulter. The exo-zodiacal light is a portion of the star light that is scattered by the micrometric dust grains in the planetary system plane plus a dust thermal emission [9]. To detect the planet transit the central star light must be suppressed through the occulter. CANYVAL-X is a NASA mission and a formation flying demonstrator. The mission consists of 2U cubesat, used for the exoplanet detection, plus a 1U cubesat, used as occulter [10]. DeMi is a MIT study about a Cubesat Deformable Mirror Demonstrator in LEO (Low Earth Orbit) to enable space-based coronagraphic direct imaging of exoplanets from a 3U cubesat platform [11]. Centaur is a pathfinder mission to directly image exoplanets, using a new kilo-deformable mirror (1024 actuators), looking at Alpha Centauri A&B. PicSat is a 3U cubesat with the purpose of monitoring Beta Pictoris, a A6V star of V = 3.86 magnitude. The payload consists of a 35 mm effective aperture objective and a single pixel avalanche photodiode. A single-mode fiber guides the star from the focal plane to the photodiode. As for an exoplanet project, a closed control loop is used to achieve a fine pointing [12].

In a mission devoted to exoplanet detection through photometric transit there are several cases in which the target star brightness decreases for reasons other than a planet transit. The false positive signal sources belong to two possible groups: hardware faults and astronomical scenarios. The second group refers to astronomical objects other than planets that transit in front of the target star. The distinctive element of a planet transit is that the star light curve decreases uniformly in the entire visible band. Unlike stars, the rocky planets temperature produces an approximately uniform spectral emission or absorption in the visible band.

The current approaches to discriminate the true signal from false positives are here briefly described.

Ground based complementary observations are often used to confirm a measurement, like photometry, high resolution spectroscopy, and radial velocity measurements [13].

Another strategy is to evaluate the pixel level data and to identify which pixels in and around the target contain the transit signal. If the event is a false positive, the pixel location of the transit signal does not coincide with the pixel location of the target flux. It works well just in cases of an eclipsing object close enough to the observed target, so that the transit signal is not too much diffused [14].

A quite demanding strategy in terms of database size is ephemerides matching, which consists of first creating catalogs of transiting planets, eclipsing binaries, and other variable stars in the instrument field of view; secondly defining a criterion to compare objects, according to their period and epochs; and then looking for objects in the catalogs that fulfill the matching criterion according to their ephemerides. Found objects indicate at least one false positive event [14].

A more recent approach is the probabilistic method, which consists of computing in a Bayesian framework the probability of the planet transit scenario against an exhaustive set of false positive scenarios. If the planet scenario is the highest probable one, then the planet is validated [13].

The estimated false detection rate of Kepler measurements is in the order of 50% (e.g., giant planet false positive rate indicated in [15]). Most of Kepler false positives are produced by background eclipsing binaries and planets transiting a star that is physically bound with the target star [13].

The false positive rate of CoRoT mission is 83%. In this mission the identification of false positives is realized only by the light-curves analysis, evaluating the transit depth and duration, the curve shape and the presence of color signature [16]. A CoRoT astronomical field is denser than Kepler, so the probability of observing a false positive transit is higher. Moreover, the CoRoT observation approach neglects the centroid follow up during the transit, i.e., measuring the centroid shift during the transit helps in rejecting possible background eclipsing binaries [13,17]. However, the main reason for this rate in CoRoT is the large PSF size of each star, such that the detector saturation was avoided but the overlapping rate of star PSFs was increased.

This project combines the growing interest in the exoplanet search with the increasing success of the cubesat platform. The project aims at detecting exoplanet photometric transit, focusing on very bright stars, and discriminating a false positive detection.

## 2. The Satellite Project

There are two key aspects when observing an exoplanet transit from space. Space-based observation ensures a high level of precision and consistency for the transit measurement, as it is required due to the tiny flux decrement (0.01%) of a Sun-like star during the transit of an Earth sized planet [1]. The reason is that observations from space do not suffer from atmospheric disturbances (seeing variations or scintillation) and limits, day-time cycle variation, moonlight, weather factors. A drawback of ground telescope observations is the limited scheduled observing time, while a space mission can be devoted even to the observation of a single target over the project lifespan. Even for ground-based instruments that are devoted to exoplanets’ search, the smallest detectable planets are Neptune-sized.

### 2.1. Mission Analysis

The project objective is to demonstrate the feasibility of a high performance astronomical mission based on a low-cost space platform, and also to show a proof of concept for a valid technique against false positive signals.

The project’s key concepts are the following.

(1) Exoplanet detection (goal Earth-like planets around Sun-like stars)
(2) Photometric transit method
(3) Identification of false positive signals
(4) 3U cubesat platform

Following a system engineering approach [18], each key concept defines a system driving requirement, which in turn determines one or more satellite subsystem requirements. The system driving requirements are shown below with the nomenclature R.x, where x is the key concept number. Table 1 summarizes the satellite subsystem requirements.

**Figure 1:**
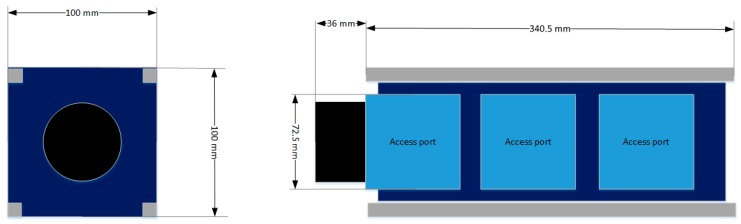
Layout of a 3U+ cubesat platform, according to standard cubesat specification.

**Figure 2:**
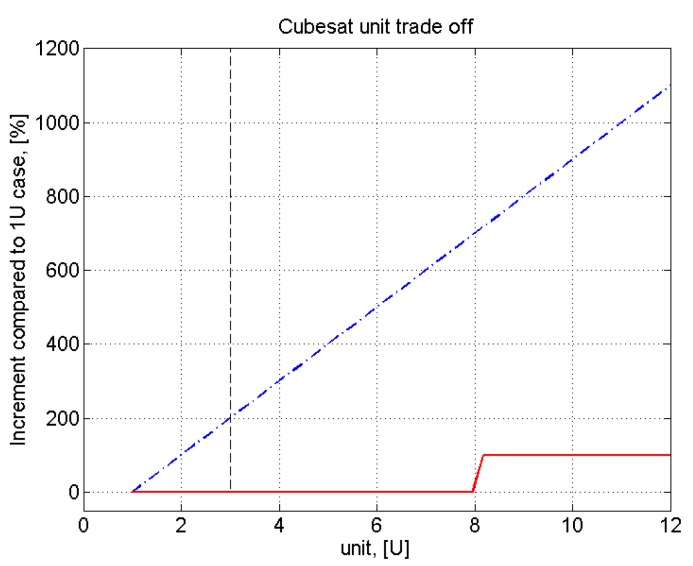
Cubesat unit tradeoff. The red line represents the available objective aperture size (D) of the payload as a function of the number of units. The blue dash-dot line is a first approximation cost (C) as a function of the unit number. The cost trend follows the linear mass increment trend as the unit number grows. Since the aperture shape in question is circular, D is unchanged until 8 units, which is the minimum number of units to build a square cubesat with a face size of 2 units. The black dashed line corresponds to the 3U cubesat size.

R.1 The amplitude of the signal to be detected is 84 ppm. During the transit the ratio between the combined flux from star and planet (F(t)) and the unobstructed star flux (Fs) is approximated by the transit depth expression δ. The transit depth δ is proportional to the square ratio between the planet radius (R_planet_) and the star radius (R_star_) [1], Equation (1) (neglecting at this time the influence of the star limb darkening on the transit depth). The assumed star is the Sun and the assumed planet is the Earth, Table 2.
  (1) $$\frac{F\left(t\right)}{F_{s}}\approx {\left(\frac{R_{\mathrm{planet}}}{R_{\mathrm{star}}}\right)}^{2}$$
  The assumed planet transit duration is 6.5 h [19]. To get a 7 σ certainty of the measured transit [20], the maximum level of tolerable noise is 12 ppm. To be conservative, this requirement is applied to the observation time equal to the duty cycle time, without taking into account the multiple transit observation. A thermal analysis will be included in the error budget, thus reducing the requirement margin. The precision of the measurement requires high-stability pointing along the entire observation. As first choice (in analogy with ExoplanetSat example) the value of 5 arcsec is needed for pointing stability.
R.2 The required payload components are the objective and the detector (photometric payload)
R.3 To discriminate the false positive signal, the photometric signal is slightly dispersed. The observation band is the visible. The required spectral resolution is low (3 bands from 400 nm to 850 nm), since the measurement purpose is to monitor the centroid location of the dispersed signal.
R.4 The standard cubesat structural limits in terms of dimensions and weight must be fulfilled. The 3U-cubesat dimensions are 10 cm × 10 cm × 30 cm (34 cm in 3U+ configuration) with a mass up to 4 kg [21], Figure 1. These small satellites require an orbital deployer (e.g., P-POD from CalPoly) to ensure that the cubesat is safely stored and correctly launched from the launcher. The advantage of choosing a cubesat platform is its straightforward design, realization, and test with a cheap budget. The drawbacks are limited size and mass, and then limited resources for power, computing, and attitude control. The technical challenge is then to fulfill the mission objective through the limited capabilities of the cubesat space platform. Moreover, the requirement of 3U size comes from the consideration that further mass increment (and thus unit increment, till about 8U) would not provide free space for an objective aperture wider than 100 mm (assuming to use a circular aperture shape). The minimum estimated size for the payload is 1 unit and at least 2 other units are required for the satellite’s subsystems. Three units is then considered the most suitable number, as seen in Figure 2.

### 2.2. Satellite Orbit and Target

Usually the selection of the orbit is influenced by multiple aspects, like the available options from the launch providers, the target position, analogy with existing missions, and considerations on the space radiation environment.

For exoplanet detection missions the orbit options depend on the platform size; for large and medium sized missions, L2 orbit and heliocentric orbits are suitable, while for medium and small platforms, a LEO orbit is common and feasible. If the satellite orbit is far away from Earth, it is possible to avoid the day–night cycle of observation. In the case of a low altitude (400 km/600 km) LEO orbit, instead, the best observing time is the orbit night, and any transit observation is possible only for an observer that lies in the penumbra of the transiting exoplanet.

The launch options for cubesats are the followings.

- as a piggyback payload from standard launchers,
- through the cubesat launch service from the ISS (Kibo module),
- through a private service from a dedicated satellite (e.g., GAUSS Unisat),
- as a primary payload from a launcher for small satellites (future option).

The launch cost range goes from 40,000 € for a piggyback launch to 150,000 € for a private satellite, or even for free in case of launch campaign for university satellites (QB50 program). Moreover, many start-up companies are building new concept rockets to launch only small satellites with a low cost and easier application process.

The assumed orbit is circular sun-synchronous at 600 km altitude and 98° inclination, as one of the available options from the launch providers (GAUSS private company, DNEPR rocket). From this assumption the computed eclipse time is 31 min over 97 min orbital period duration. Further analyses will be conducted to identify the requirements about the straylight during the transit observation. These future considerations could affect the orbit altitude and platform orientation options.

The number of known dwarf stars (luminosity class V) of V-magnitude less than four is 115 (91 dwarfs have V-magnitude less than 3.8) (From SIMBAD astronomical database, http://simbad.u-strasbg.fr/simbad/). Currently (February 2017) there are four confirmed exoplanets orbiting dwarf stars of V-magnitude less than 4, and six unconfirmed exoplanets orbiting around the same type of star (From http://exoplanet.eu/). None of them has been ever observed with the photometric method from space. One of the confirmed exoplanet host stars is Alpha Centauri B (planet Alpha Centauri Bb), and further investigations are required for the star companion Alpha Centauri A. The first option target is Alpha Centauri AB, which is the closest-to-the-Sun binary star system that could host a planet in the habitable zone, and it is the most feasible target to be observed from a small platform [22]. Alpha Centauri A’s V-magnitude is 0.01, and Alpha Centauri B’s V-magnitude is 1.33. It is still to be defined the observing strategy, e.g., switching from one star to the companion, or choosing one of the two as a permanent target.

### 2.3. Cubesat Design

The cubesat platform consists of several subsystems. The payload subsystem will be the main focus of this paper, and a preliminary design for the other subsystems will be described.

**Figure 3:**
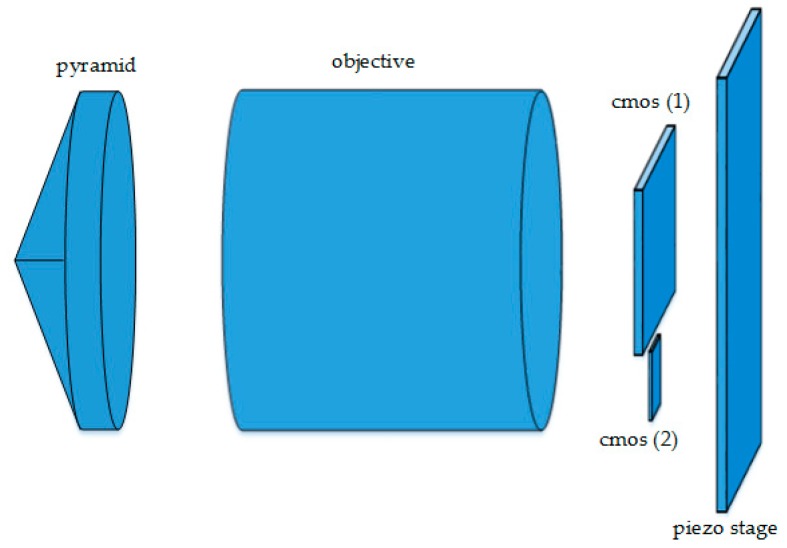
Payload concept.

At the present design stage, the payload consists of the following parts, as seen in Figure 3.

**Figure 4:**
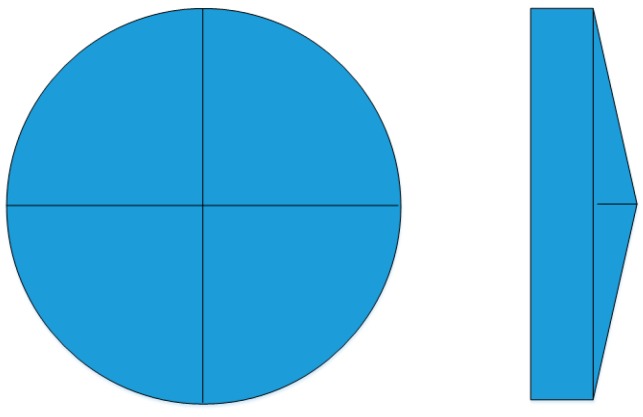
The four-facet round-base pyramid concept.

- a glass pyramid, with a round base and four facets, as seen in Figure 4,
- a commercial objective,
- a scientific detector, cmos (1),
- a second detector used to close the attitude control loop, cmos (2),
- a two-axis piezo-stage behind the detectors to compensate for the spacecraft jitter in a closed control loop.

The pyramid is located before the objective. The four pyramid facets form four images, and the four sky fields are overlapped in the sky area including the target star. The four images of the target star and surrounding sky field identify four detector areas, in which the photometric measurements take place. These are the so-called detector photometric windows, whose size can be adjusted according to the available data rate.

The pyramid allows for four simultaneous recordings of the transit, so that the overall transit measurement is free from instrumental fault events due to the detector. The probability (P4) that an instrumental fault event happens in all of the four windows is equal to the fourth power of the probability (P1) of an instrumental fault in one window; thus, P4 is greatly lower than P1.

**Figure 5:**
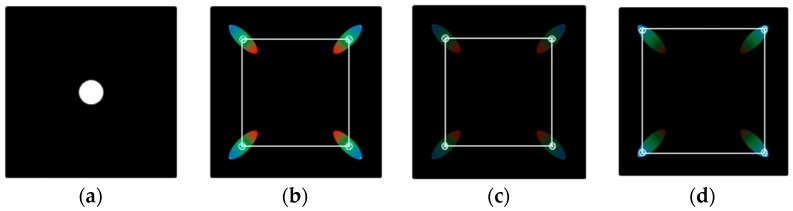
Principle of measurement: (**a**) shows the ideal spot image of a star, as seen through the payload without the pyramid; (**b**) shows the ideal spot image of a star as seen through the payload with the pyramid; the four spots are dispersed and the centroids (white circles) are joined by white lines to show their relative distances; (**c**) shows the case of image (**b**) during the transit of a planet, a uniform decrement of luminosity determines no shift of centroids; (**d**) shows the case of image (**b**) during a false positive transit, where the non-uniform decrement of luminosity determines the shift of centroids and then the change of their relative distances.

The pyramid also has a dispersive power, and its spectral resolution is determined by the pyramid base angle. The dispersion direction in each photometric window is rotated 90° with respect to the neighboring photometric windows. Before and during the transit the centroid of the photometric signal is computed in each window. If a planet transit occurs, the signal reduction is spectrally uniform and the relative distance between the windows centroids is kept constant. In case of an astronomical false positive event, the transit could be made by the star companion of a binary system, and the signal reduction is not spectrally uniform, due to the transiting star spectral emission and absorption. In this case the relative distances between the window centroids change, as seen in Figure 5.

The pyramid material first option is the commercial BK7 glass; a radiation resistant glass is also considered, i.e., BK7 G18 SCHOOT, and no pyramid design variation is required.

**Figure 6:**
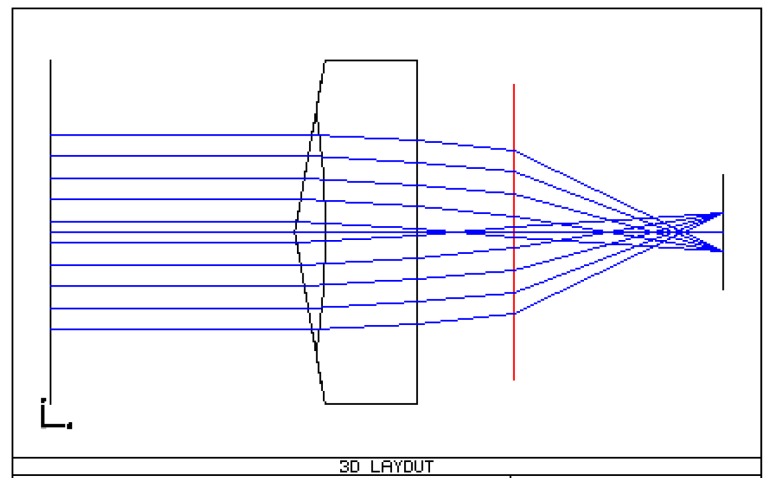
First pyramid design in zemax. The red line is the equivalent lens corresponding to the objective.

The design of the pyramid consists of the definition of the pyramid base angle according to the required rays deviation and image dispersion. To avoid saturation and to have a broad detector dynamic range, the star spot is defocused. The star defocus is assumed to be 10 × 10 pixels; a simplified sketch of the optical system has been realized in ZEMAX, thanks to the custom zemax library PAM2R [23], as seen in Figure 6. Other assumptions are: the photometric window size is 40 × 40 pixels, and the ray deviation from the optical axis is 300 pixels, in the two perpendicular directions on the detector plane. From geometric considerations the angular deviation α is 3.64°. Considering the material reference refractive index *n* equal to 1.52, and according to the simplified Equation (2) [24], the pyramid base angle θ is 7°.

Considering the refractive index variation in the band from 410 nm to 850 nm, each star spot is dispersed over 11 pixels, so that a bandwidth of 150 nm corresponds to 3.5 pixels. Adding in quadrature the defocus and the dispersion contribution, the total elongated star dimension is 15 pixels.

(2) θ=α (n−1)

The piezoelectric stage is located behind the focal plane. It is the key element to reduce the spacecraft jitter to few ppm of noise. The stage works in a closed loop that keeps the target image on the same pixels during the scientific exposure. The detector involved in the closed loop is an auxiliary sensor with a pixel size smaller than the scientific detector pixel size, and thus it reaches a higher frame rate. According to [25] this technique (together with the MIT driver code) let to reach the pointing accuracy of 2.3 arcsec.

The first stage option is a custom Physik Instrumente (PI) stage model, with custom changes to fit the cubesat platform. The two axes nanometric PI stage, PI P-733.2, has a travel range of 100 µm (for each axis) and a resolution of 0.1 nm. Another option is XY200M, a two axes piezo stage from CEDRAT Technologies, with a travel range of 200 µm (no load value) and a resolution of 20 nm.

The considered commercial objective is the ZEISS Planar T 1.4/85, with a focal length of 85 mm and the aperture diameter of 60.7 mm. The projected image on the focal plane has a diameter of 43 mm [26]. The objective has been tested for space stresses through a scientific research work from ZEISS laboratory [27].

The selected scientific detector is the HAS2 image sensor. It is the High Accuracy Star tracker CMOS image sensor from ON Semiconductor. A second detector is used to achieve a fine attitude control (e.g., e2V EV76C454 CMOS sensor). The star tracking activity is performed from the HAS2 sensor on the image resulting from the pyramid and the objective combination, and thus the adoption of a cross-correlation algorithm is foreseen. The HAS2 features are listed in Table 3. The HAS temperature features are: dark current doubling for sensor temperature increment of 5.8 °C (average), and voltage-temperature variation of −4.64 mV/°C. If needed, temperature control based on a Peltier module will be considered. The HAS2 sensor has been tested for functionatility up to 300 krad, and up to 42 krad the functionality is guaranteed. Additional considerations and detailed studies on this topic will be carried out in the next steps of the project. However, even at this stage, it can be seen that this sensor is widely used in space applications such as star sensors for attitude determination whose lifetime is several years. The latter performance, even scaled down at the level of a cubesat system, will be in excess of the cubesat satellite’s nominal lifetime.

The payload subsystem should fit in one cubesat unit, that is in a 100 × 100 × 120 mm^3^ cube, and should weight no more than 1330 g. The pyramid base height is assumed as 10 mm and the pyramid height is 3.8 mm. Table 4 lists the sizes and weights of each payload component; each X- and Y- size must be less than or equal to 100 mm, the sum of Z sizes must be less than or equal to 120 mm, and the sum of the weights must be less than 1330 g.

The attitude determination and control consists of an off-the-shelf module with at least 3 reaction wheels and 3 axis magnetometers, for instance MAI-400 from Maryland Aerospace. The attitude determination is mainly achieved through the star tracker camera HAS2. The fine target pointing is achieved through a closed loop control system realized with a nanometric 2-axis stage and a second auxiliary detector (e.g., PI stage and e2V EV76C454 CMOS, as described above).

The on-board scientific and attitude data handling will be realized through a commercial off the shelf single board computer suitable for cubesats.

The electrical power is supplied by deployable solar panels, and the likely configuration option is to deploy the panels and to arrange them in one single plane. The thermal control will be passive.

## 3. Star Visual Magnitude Limit

The most demanding requirement refers to the capability of detecting the signal with amplitude equal to 84 ppm and with a maximum noise level of 12 ppm. There is a limit in the target star V-magnitude, below which this requirement is fulfilled. This limit is computed in the following steps.

The first step is the evaluation of the photon flux from typical target stars. The considered V-magnitude (vmag) range is from 0 to 5. The photon flux (s_flux_) is given in photons per seconds, scaling the reference photon flux of Vega in the visible band. The assumed optical aperture is the one from Zeiss objective (2893 mm^2^).

The overall signal level is then computed in terms of detected photons, considering a range of possible exposure time (t_exp_) from 0.01 s to 10 s, and of a number of co-added frames (#Frames) from 1 to 10,000, Equation (3).

The second step is the noise computation. The noise sources taken into account are: photonic noise (N_phot_), dark current noise (N_dark_), and read out noise (N_ron_) [28], Equations (4)–(6). The detector choice sets the dark current value (I_dark_), expressed in electrons per pixel per seconds, and the read out characteristic (RON), expressed in electrons per pixel.

The third step is the S/N ratio computation, Equation (7). The dominant noise source is the photon noise, and the S/N can be approximated as the square root of the signal, Equation (8). The requirement of maximum noise level of 12 ppm corresponds to a S/N of 10^5^, and requires a star signal level of 10^10^ photons. The star spot is intentionally defocused, and the star photons are spread on 10 × 10 pixels, leading to 10^8^ photons per pixel required.

(3) $$s=s_{\mathrm{flux}}\times t_{\exp}\times \#\mathrm{Frames}$$

(4) $$N_{\mathrm{phot}}^{2}=s$$

(5) $$N_{\mathrm{dark}}^{2}=I_{\mathrm{dark}}\times t_{\exp}\times \#\mathrm{Frames}\times \#\mathrm{pixel}$$

(6) $$N_{\mathrm{ron}}^{2}={\mathrm{RON}}^{2}\times \#\mathrm{pixel}\times \#\mathrm{Frames}$$

(7) $$\frac{S}{N}=\;\frac{s}{\sqrt{N_{\mathrm{phot}}^{2}+N_{\mathrm{dark}}^{2}+N_{\mathrm{ron}}^{2}}}$$

(8) $$\frac{S}{N}\approx \;\sqrt{s}\;(\text{If readout noise and dark noise are negligible})$$

The constraints related to the achievable S/N are the following, shown in Table 5.

- The observation time on each orbit (i.e., the product between the exposure time of a single frame and the number of co-added frames) must be less than or equal to the duty cycle time. The total transit observations should include one full transit time before and after the transit, to better estimate the transit baseline.
- The exposure time of each frame must be less than the time required to saturate the detector. This constrain is expressed as the number of photons corresponding to the saturation voltage, taking into account the detector conversion factor and the quantum efficiency.

The next computation steps, from number 4 to number 8, identify the star magnitude limit according to the constraints (see Table 6).

**Figure 7:**
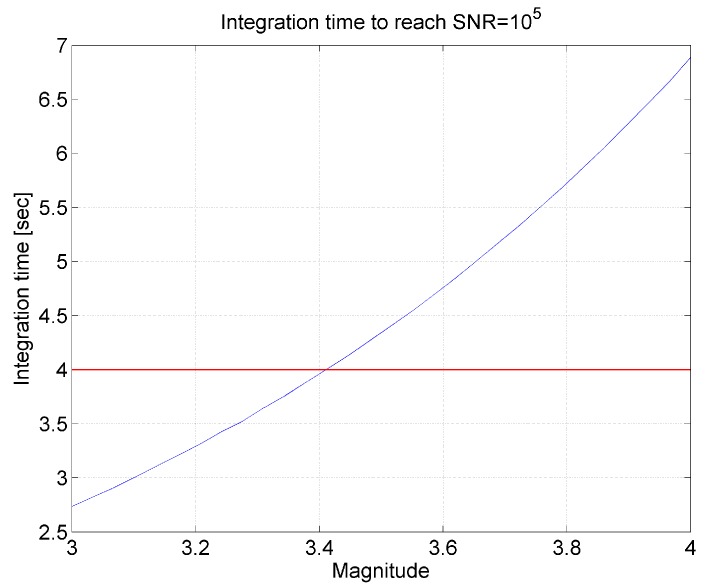
Integration time as function of target star V-magnitude. The SNR is 10^5^. The red line is the maximum integration time limit, i.e., 4 s.

The results of the computation show that star V-magnitude equal to 3 is the limit, since the observation of a star of fourth V-magnitude would require an exposure time longer than 4 s. A finer analysis in the magnitude range from 3 to 4 shows that the limiting V-magnitude is 3.38 (Figure 7).

The data rate computation is relative to four 40 × 40 photometric windows and a 12 bit resolution detector; for a target star of V-magnitude 3 (i.e., 453 frames per orbit) the data rate is 34.8 Mbit per orbit. The data will be stored, waiting for the ground station to be in view.

## 4. Discussion and Conclusions

Payload design for exoplanet transit detection from a 3U cubesat platform is a challenging mission, but it is consistent with the trend of using cheaper space platforms for highly demanding scientific missions. The objective of the project is to analyze the feasibility of this kind of mission, and also of using the pyramid to provide measurements free of false positives.

The 3U cubesat transversal size limits the maximum aperture of the objective, and this affects the V-magnitude of the fainter observable star. From the approximated computations illustrated above, the possible targets are very bright stars with V-magnitudes equal to or lower than 3.38. Even though this is a tight range of possible targets, photometric transit observation from space has never been done for these bright targets.

The attitude subsystem and the target pointing are essential in all the demanding applications for cubesats, especially for astronomical missions. The drawback of aiming at these high-performance objectives is that much customization could be required, which cancels out the low-cost advantage of the cubesat project. The attitude and pointing subsystem could be the subject of future research activities.

## Figures and Tables

**Table 1:** Matrix of subsystem requirements.

Subsystem	R.1	R.2	R.3	R.4
*Cubesat Payload Subsystem*	The signal amplitude is 84 ppm, the tolerable noise level is 12 ppm in a duty cycle time observation. The detector resolution should be greater than or equal to 12 bit.	The payload is a photometric payload (at least objective + detector) in the visible band.	The transit measurement signal must be redundant and spectrally dispersed.	The optical aperture diameter should be less than 10 cm.
*Cubesat Attitude determination and Control*	High-stability pointing should be provided to detect the signal decrement (5″).			Total stored chemical energy will not exceed 100 Watt-hours.
*Cubesat Command and data handling*	The onboard processing should be as limited as possible, to avoid any data alteration.		Select the scientific data to be stored according to the available data rate.	Total stored chemical energy will not exceed 100 Watt-hours.
*Cubesat Structure and Mechanism*		No more than one unit free space is available for the payload subsystem.		Platform dimensions are 10 cm × 10 cm × 34 cm. Platform maximum mass is 4 kg.
*Mission Operations*	The minimum mission lifetime is four years, to measure the transit of an exoplanet with an orbital period of one year at least three times.	The scientific operations consists in looking at one target star continuously to measure the star flux along the entire orbital period of the exoplanet.		

**Table 2:** Sun and Earth radius, expected transit depth of an Earth-like planet around a Sun-like star.

Parameter	Value
R_star_, Sun volumetric mean radius (km)	695,700
R_planet_, Earth volumetric mean radius (km)	6371
δ, transit depth	84 × 10^−6^

**Table 3:** List of main characteristics of HAS2 star tracker and ZEISS Planar T 1.4/85 objective.

HAS2		ZEISS Objective	
Parameter	Value	Parameter	Value
Overall dimensions	30 mm × 30 mm	f	85 mm
Image sensor format	1024 × 1024 pixels	F/#	1.4
Pixel size	18 μm	Diameter	60.7 mm
ADC resolution	12 bit	Image diameter	43 mm
Saturation voltage output	1.49 V	FOV	12.42 deg
Full well capacity	10^5^ e	Pixel scale	44.15 arcsec/pix
Quantum Efficiency	45% (500–650 nm)		
Spectral response	33.3% (400–900 nm )		
Conversion factor	14.8 µV/e		
Dark current	12.5 e/pix/s		
RON	2 e/pix		

**Table 4:** List of payload components and budget of dimensions and weight for the payload unit.

Component	Name	Length X (mm)	Length Y (mm)	Length Z (mm)	Weight (g)
Pyramid	custom, BK7 glass	62	62	13.8	85.38
Objective	Planar T 1.4/85 ZF	77	77	62	570
Detector	HAS2	30	30	4.5	8
Detector	e2V	10	10	2	5
Imager board	custom PCB				3
Stage	PI P-733.2 CL	100	100	25	580
Stage controller	custom (on PCB)				3
Check/Tot		each ≤ 100	each ≤ 100	107.3	1254.38

**Table 5:** Constraints, requirements, and assumptions for the limiting V-magnitude computation.

Constraints		Requirements		Assumptions	
Duty cycle time [min]	31	S/N	≥10^5^	window [pix]	40 × 40
Photons to saturate [phot/pix]	2.237 × 10^5^	Photons/pix	10^8^	system efficiency	70%
				star defocus [pix]	10 × 10

**Table 6:** Computation steps to determine the limiting V-magnitude.

Computation Steps			
Step	Step Title	Step Description	Values
1	Compute the signal level	(*see the text above*)	
2	Compute the noise level	(*see the text above*)	
3	Compute the S/N ratio	(*see the text above*)	
4	Min number of frames	Photon per pixel/Photon to saturate	10^8^/(2.237 × 10^5^) = 447 frames
5	Max exposure time per frame	Eclipse time/Min number of frames	4 s
6	Exposure time limit (t_lim_) to avoid saturation	Compute the detector voltage corresponding to the incoming photon flux for one frame. Find the exposure time to get the higher voltage value without saturation. The star flux is spread on 10 × 10 pix.	vmag = 0, t_lim_ = 0.17 s; vmag = 1, t_lim_ = 0.43 s; vmag = 2, t_lim_ = 1.09 s; vmag = 3, t_lim_ = 2.74 s; vmag = 4, t_lim_ = 6.89 s; vmag = 5, t_lim_ > 10 s;
7	Number of co-added frames	Compute the number of co-added frames required to get a S/N of 10^5^ (star signal of 10^10^).	vmag = 0, frames = 451; vmag = 1, frames = 451; vmag = 2, frames = 451; vmag = 3, frames = 453; vmag = 4, frames = 461; vmag = 5, frames = 823 (10 s);
8	Verify the compliance with the duty cycle time constraint.	t = frames × tlim	vmag = 0, t = 77.75 s; vmag = 1, t = 194.94 s; vmag = 2, t = 492.43 s; vmag = 3, t = 1243 s; vmag = 4, t = 3175 s; vmag = 5, t = 8236 s.

## Abbreviations

FOV	Field of view
MIT	Massachusetts Institute of Technology
NASA	National Aeronautics and Space Administration
ESA	European Space Agency
CNES	Centre national d’études spatiales
TESS	Transiting Exoplanet Survey Satellite
CHEOPS	CHaracterizing ExOPlanets Satellite
PLATO	PLAnetary Transits and Oscillations of stars
CoRoT	Convection, Rotation and planetary Transits
HEO	High Earth Orbit
LEO	Low Earth Orbit
CCD	Charge Coupled Device
PI	Physik Instrumente
ppm	parts per million
S/N	Signal to Noise ratio
vmag	visual magnitude
